# Colors of Single‐Wall Carbon Nanotubes

**DOI:** 10.1002/adma.202006395

**Published:** 2020-12-14

**Authors:** Nan Wei, Ying Tian, Yongping Liao, Natsumi Komatsu, Weilu Gao, Alina Lyuleeva‐Husemann, Qiang Zhang, Aqeel Hussain, Er‐Xiong Ding, Fengrui Yao, Janne Halme, Kaihui Liu, Junichiro Kono, Hua Jiang, Esko I. Kauppinen

**Affiliations:** ^1^ Department of Applied Physics Aalto University School of Science Aalto 00076 Finland; ^2^ Department of Physics Dalian Maritime University Dalian 116026 China; ^3^ Department of Electrical and Computer Engineering Rice University Houston TX 77005 USA; ^4^ School of Physics Academy for Advanced Interdisciplinary Studies Collaborative Innovation Center of Quantum Matter Peking University Beijing 100871 China; ^5^ Department of Physics and Astronomy Rice University Houston TX 77005 USA; ^6^ Department of Materials Science and NanoEngineering Rice University Houston TX 77005 USA

**Keywords:** carbon nanotube thin films, color range, nanomaterials, pigments, quantitative coloration model

## Abstract

Although single‐wall carbon nanotubes (SWCNTs) exhibit various colors in suspension, directly synthesized SWCNT films usually appear black. Recently, a unique one‐step method for directly fabricating green and brown films has been developed. Such remarkable progress, however, has brought up several new questions. The coloration mechanism, potentially achievable colors, and color controllability of SWCNTs are unknown. Here, a quantitative model is reported that can predict the specific colors of SWCNT films and unambiguously identify the coloration mechanism. Using this model, colors of 466 different SWCNT species are calculated, which reveals a broad spectrum of potentially achievable colors of SWCNTs. The calculated colors are in excellent agreement with existing experimental data. Furthermore, the theory predicts the existence of many brilliantly colored SWCNT films, which are experimentally expected. This study shows that SWCNTs as a form of pure carbon, can display a full spectrum of vivid colors, which is expected to complement the general understanding of carbon materials.

## Graphical abstract

The colors of 466 species of single‐wall carbon nanotubes (SWCNTs) are revealed by experiments on clean dry thin films and modeling of the carbon nanotube absorbance spectra. A quantitative coloration model of SWCNTs is proposed and is verified by existing experimental data. This model predicts the existence of brilliantly colored carbon nanotube films that can display the full rainbow.

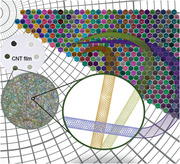

Carbon materials generally appear either colorless or black. For instance, a chemically pure and structurally perfect sp^3^‐bonded diamond is totally transparent and shows no color, while sp^2^‐bonded graphitic structures, such as graphite, graphene,^[^ ^1^, ^2^ ^]^ and carbon nanotubes (CNTs)^[^ ^3^, ^4^ ^]^ are usually black. CNTs are commonly understood to comprise carbon atoms in a hexagonal, honeycomb‐like lattice, shaped into a tubular structure. In particular, as‐produced multiwall CNTs are even recognized as super black material,^[^ ^5^ ^]^ which can be utilized in optical systems and space applications.^[^ ^6^ ^]^ However, later studies have shown that when single‐wall CNTs (SWCNTs) are sorted by diameter^[^ ^7^, ^8^, ^9^, ^10^ ^]^ or atomic structure [i.e., (*n*,*m*)],^[^ ^11^, ^12^, ^13^, ^14^ ^]^ they strikingly exhibit various distinct colors in suspension. More recently, colorful SWCNT thin films were produced by a novel one‐step fabrication procedure,^[^ ^15^ ^]^ enabling the direct synthesis of colorful SWCNTs that were previously thought impossible.

Despite numerous studies on SWCNT colors, the coloration mechanism has not been fully understood. Currently, no theoretical model can reliably predict the color of a given SWCNT film. Namely, predicting the range of possible SWCNT colors has not been possible, nor has explaining why SWCNTs with certain atomic structures [specified by (*n*,*m*)] display stronger colors than other CNT species. This lack of understanding largely prevents us from further elaborating the controlled synthesis of color‐specified SWCNT materials.

Here, we report the results of both experimental and theoretical studies providing answers to these long‐standing questions. First, by studying colored thin films with varying thicknesses, we established a quantitative relationship between the obtained colors and the optical absorption spectra of the films. This relationship confirms the absorption‐dominated coloration mechanism of SWCNT‐based films. Based on this finding, in combination with SWCNT optical absorption studies, we constructed a comprehensive theoretical model that can describe and predict colors for various species of SWCNTs with different (*n*,*m*). We performed calculations for 466 different (*n*,*m*) species of SWCNTs and found excellent agreement between the theoretically calculated and existing experimental data. Our results will enable the further development of permanent pure‐carbon dyes^[^ ^8^ ^]^ as well as electrochromic devices^[^ ^9^ ^]^ for optical and aesthetic applications.

To discover the relationship between the optical absorption spectrum (OAS) and visual color, we studied dry film samples with various thicknesses. **Figure** **1**a presents photographs of six samples prepared under the same synthesis conditions^[^ ^15^ ^]^ but with different collection durations, which determine the thickness of the film. The photographs are uniquely calibrated (Figure S1, Supporting Information) so that the color obtained from the calibrated photographs is reproducible within the precision of human eyes,^[^ ^16^, ^17^ ^]^ regardless of variations in the camera and lighting settings. Figure 1a shows that the same (*n*,*m*) composition^[^ ^15^ ^]^ can exhibit different colors at different thicknesses. The thin (10‐min sample) and relatively thicker film (150‐min sample) are less obviously green than the samples of moderate thickness. Notably, previous studies have demonstrated that to reliably extract color from images, lighting control and photographic calibration are required.^[^ ^18^ ^]^ The careful photographic calibration in this study ensures experimental repeatability and provides a foundation for further analysis.

**Figure 1:**
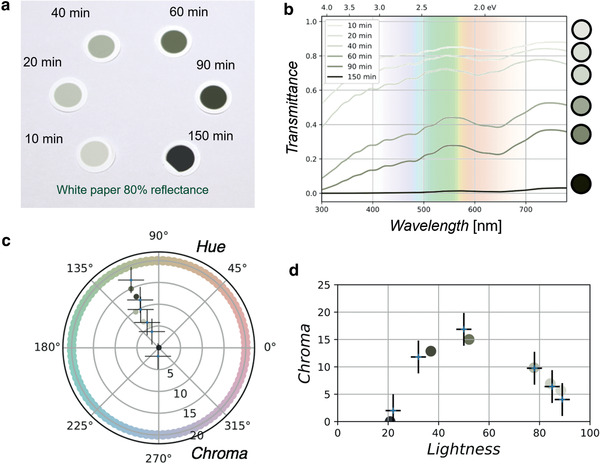
Relationship between collection time and color of film. a) Calibrated photographic image of as‐synthesized SWCNT films with six different thicknesses indicated by the duration of collection. The colors range from light gray to obviously green to nearly black. b) Transmittance spectra of the same SWCNT films. Right circles: colors calculated from each corresponding spectrum. c) Top view of the perceptually uniform lightness–chroma–hue space (L*C*h). Color coordinates measured from the calibrated photographs (+) and calculated from the OAS (•). Error bars indicate uncertainty Δ*E* ≈ 5 for measuring colors in the calibrated photograph. In the top view where the radial (circumferential) direction indicates chroma (hue), the six colors lie on the same line pointing toward the green–yellow hue, which indicates that varying the film thickness does not change the hue. d) Side view of the L*C*h space. The maximum chroma (most vivid color) is obtained at a moderate absorbance, whereas films that are either too thin or too thick are less visually colorful.

The OAS of the samples were studied by directly transferring the photographed films from the white filters to quartz substrates, which were then characterized in a spectrophotometer. Figure 1b shows the acquired OAS of the six samples shown in Figure 1a in terms of the transmittance spectra *T*(λ) = 10^−*A*(λ)^, where *A*(λ) is the measured optical absorbance, and *T*(λ) is the transmittance. The circles on the right side of Figure 1b indicate colors calculated from the acquired OAS using a modeled relationship (see Methods in the Supporting Information) and the standard quantitative process.^[^ ^19^, ^20^, ^21^ ^]^ Although OAS is widely used to characterize colorful SWCNT‐based samples,^[^ ^7^, ^10^, ^11^, ^15^, ^22^ ^]^ no quantitative relationship has been established between the OAS and the color properties of SWCNTs. The coloration of SWCNT suspensions can be explained qualitatively by subtractive color theory^[^ ^10^ ^]^ as the color of SWCNTs is dominated by absorption, like pigments. The same trend can be observed in the presented dry film samples. As shown in the transmittance spectra of Figure 1b, the greenish‐yellow color of the film can be explained by the fact that the film allows more yellow and green light to pass through than any other color of light. We note that the absorption‐dominated nature of SWCNT color is also attributed to the weak scattering factor of SWCNT films^[^ ^23^ ^]^ and their luminescence, which lies outside the visible region.^[^ ^22^, ^24^ ^]^ Comparing Figures 1a and 1b reveals excellent agreement between the colors measured from the calibrated photograph and those calculated from the acquired OAS.

The relationship between the visual color and the OAS of an SWCNT film can be further studied by a standard analytical method. In order to ensure comparability, the color coordinates measured from the calibrated photograph and those calculated from the OAS are decomposed into three scalar components, namely, lightness, chroma, and hue (i.e., in the CIE L*C*h color space).^[^ ^16^ ^]^ Figure 1c,d show the top and side profiles of the decomposed 3D (Figure S2, Supporting Information) color coordinates, respectively. While the lightness indicates the amount of light relative to that of the reference (zero absorbance corresponds to pure white, i.e., lightness = 100, in this study), chroma and hue indicate the vividness and the type of the color, respectively. Compared with other scales such as luminance–hue–saturation, this more psychovisual linear scale enables a more accurate quantification that better matches the perception of human eyes.^[^ ^16^ ^]^ In both Figures 1c and 1d, the measured and calculated colors of each sample correspond well within the error range, suggesting the validity of this model for calculating the color of SWCNTs from their OAS. As shown in Figure 1c, the data points of SWCNT films with various absorbance values lie on a line of constant green‐yellow hue, indicating that SWCNT films with different thicknesses exhibit the same hue. The change in chroma is more informative. As shown in Figure 1d, one film exhibits an optimal absorbance to display the most vivid color, indicated by the highest chroma (Table S1, Supporting Information). This property provides a fair way to compare the colorfulness of different SWCNT species. Given an OAS of a certain SWCNT sample, we can attain the most vivid color by reliably simulating its OAS for different thicknesses according to the Beer–Lambert law.^[^ ^25^, ^26^, ^27^ ^]^ A similar trend can be expected for SWCNT suspensions, where the absorbance is tuned by varying the length of the optical path or by varying the SWCNT concentration.

**Figure 2:**
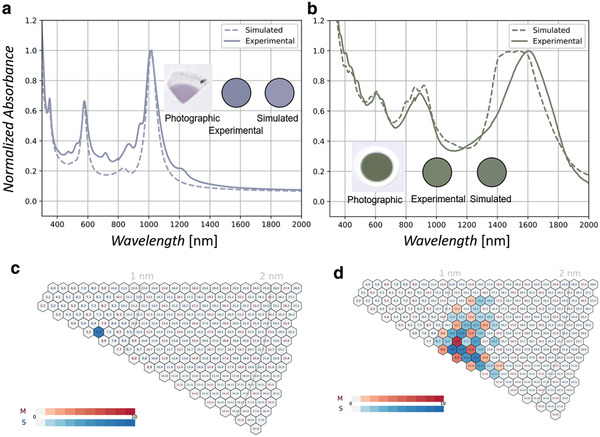
Verification of the coloration model by SWCNT film results. a) Simulated OAS of (6,5) SWCNT film and measured OAS of solution‐separated (6,5)‐SWCNT‐enriched film formed by vacuum filtration. Inset: photograph of the film on filter paper and colors calculated from the experimental and simulated OAS. b) Experimental and simulated OAS of FC‐CVD SWCNT dry film with many (*n*,*m*) species. Inset: photograph of the green film (identical to the 60 min sample of Figure 1a), calculated color from the experimental OAS, and color calculated by adding up the simulated OAS generated from optical transitions. c) (*n*,*m*) composition for simulating the OAS of (a), which is purely (6,5) SWCNTs. d) (*n*,*m*) composition for simulating the OAS of (b), which is obtained by electron diffraction in a transmission electron microscope.^[^ ^15^ ^]^ In the legend, M and S represent the metal and semiconductor species, respectively, and the graded color represents the count of each (*n*,*m*) in the sample statistics.

Since we have established the correlation between the OAS and color of SWCNT samples, we move further to establish a general coloration model (see Methods in the Supporting Information and Figure S3, Supporting Information) that describes the relationship between color and the atomic structure [i.e., (*n*,*m*)] of SWCNTs. This coloration model has four modularized sections. The first section (the Transition sub‐model) is for simulating SWCNT optical transition energies^[^ ^25^, ^28^ ^]^ based on an extended tight‐binding calculation with many‐body corrections.^[^ ^29^, ^30^, ^31^, ^32^ ^]^ The second section (the Absorbance sub‐model) is based on a systematic model for the SWCNT absorption cross‐section^[^ ^33^, ^34^ ^]^ (see Methods in the Supporting Information and Figure S4, Supporting Information). The next sections (the Radiance and Coordinate sub‐models) are based on the aforementioned OAS calculations and color standards, respectively. To demonstrate how the coloration model works, we first produced a solution‐filtrated film enriched with (6,5) SWCNTs.^[^ ^35^, ^36^, ^37^, ^38^ ^]^ We then compared the OAS and its color calculated using the coloration model (**Figure** **2**a). The spectra were normalized by the first transition (*S*_11_) peak. A π‐plasmon peak fitted with a Lorentzian shape was included as a routine.^[^ ^28^, ^34^, ^39^ ^]^ Figure 2a presents the calibrated photographic image, the color calculated from experimentally acquired OAS, the color calculated from simulated (6,5)‐SWCNTs, and the spectra are in good agreement. Note that the spectrum generated by the absorbance sub‐model in conjunction with the fit of the plasmon peak accounts for the relative peak height and width in the dry SWCNT film, thus offering a more accurate prediction than other existing models.^[^ ^28^, ^34^, ^39^ ^]^ In this way, the coloration model effectively explains and predicts the OAS and colors of (*n*,*m*)‐SWCNT‐enriched films.

The coloration model was applied to calculate the color of SWCNTs with specific (*n*,*m*). **Figure** **3**a shows the color map of a total of 466 (*n*,*m*) SWCNTs. Most species [e.g., (15,7) and (16,1)] display vivid colors, while the color of a few other species [e.g., (18,11) and (20,10)] appears grayish. We note that as the diameter increases, the colors of SWCNTs tend to have lower chroma (appear less vivid), similar to the trend of large‐diameter SWCNTs approaching the character of graphene (Figure S5, Supporting Information). These 466 (*n*,*m*) combinations cover SWCNTs that were experimentally separated^[^ ^11^, ^12^, ^13^, ^14^, ^40^ ^]^ or identified.^[^ ^15^, ^33^, ^41^ ^]^

**Figure 3:**
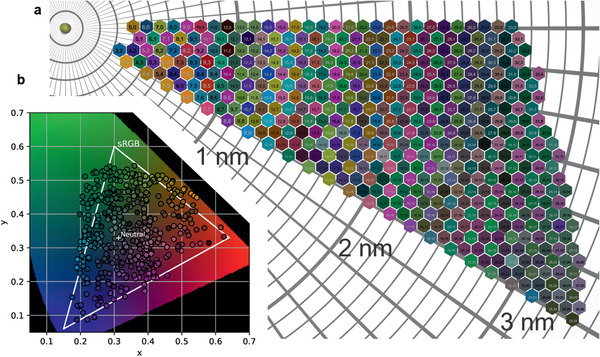
Relationship between the colors and (*n*,*m*) of SWCNTs. a) Map of the most vivid colors of 466 (*n*,*m*) CNT species. The smaller‐diameter species tend to have higher chroma (more vivid colors). b) The calculated colors plotted on the CIE chromaticity diagram compared with the sRGB gamut (color range).

To illustrate the color range of SWCNTs, the color map in Figure 3a is plotted on the CIE chromaticity diagram in Figure 3b,^[^ ^19^ ^]^ wherein the triangle shows the color range of standard red–green–blue (sRGB) displays. Pioneering work by Yanagi et al. has shown that SWCNTs can form primary colors,^[^ ^9^ ^]^ which can be mixed to obtain colors of arbitrary hue. Here, the coloration model further indicates how high the chroma of the colors can reach, which is important for real applications. Some SWCNT species with extreme chroma in the red region are (9,3) and (8,5). Some examples in the green region are (18,5) and (22,0) SWCNTs (Figure S6, Supporting Information). Some of the points are located outside the sRGB color range (Figure 3b), meaning that the colors of these SWCNTs are so intense that they exceed the color display capacity of standard display screens. Although most of those colorful SWCNT species have not been experimentally prepared, our model predicts their existence; thus, we predict a future in which vividly colored SWCNT thin films or suspensions are experimentally available.

The calculated colors of SWCNTs with different (*n*,*m*) can be verified with existing experimental data. **Figure** **4** displays photographs of single‐(*n*,*m*)‐enriched SWCNT suspensions from various experimental studies.^[^ ^11^, ^12^, ^13^, ^14^ ^]^ These photographs, without color modification, are directly compared with the calculated results from the coloration model. A minor mismatch can be observed between experimental samples of the same (*n*,*m*), especially for popular (*n*,*m*) for which data are available from different references. The chroma and lightness vary from sample to sample, but the hues generally match. For example, with (7,5) SWCNTs, which were present in all four references, the aqueous two‐polymer phase‐extracted sample (Figure 4d) has a fainter color than the other three samples. Nevertheless, all four (7,5) samples exhibit an aquamarine (light blue‐green) hue, in agreement with the color calculated for that (*n*,*m*) (Figure 4e). We ascribe such minor mismatch to different sample concentrations, lighting and photographic conditions, and minor impurities in the sample. Nevertheless, the excellent match between the prediction of the coloration model and the experimental results of solution‐processed SWCNTs suggests that the (*n*,*m*) composition mainly accounts for the color of SWCNT‐based samples. The color map (Figure 3a) generated by the coloration model can also explain the colors of samples based on mixtures of multiple (*n*,*m*) SWCNTs (Figure S7, Supporting Information).^[^ ^7^, ^8^, ^9^, ^10^ ^]^

**Figure 4:**
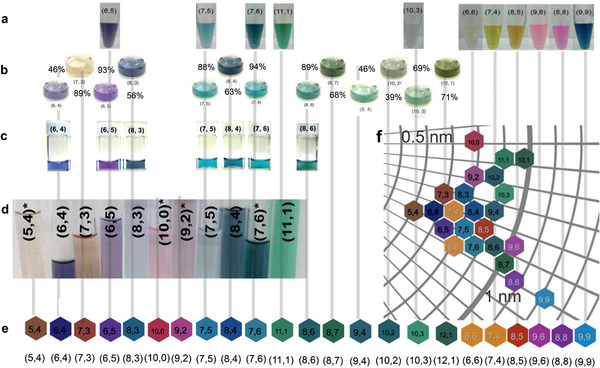
Verification of calculated colors by experimental images of single‐chirality‐enriched SWCNT suspensions. Images are cropped and moved for convenience of comparison, but their colors are not altered. a–d) Comparison of: a) aqueous two‐phase separation by DNA; b) simple gel chromatography; purities from the original report are indicated by the percentages; c) temperature‐controlled gel chromatography; d) aqueous two‐phase extraction separated semiconducting CNT suspensions. a) Adapted with permission.^[^ ^13^ ^]^ Copyright 2016, American Chemical Society. b) Adapted with permission.^[^ ^11^ ^]^ Copyright 2011, Springer Nature. c) Adapted with permission.^[^ ^14^ ^]^ Copyright 2013, American Chemical Society. d) Adapted with permission.^[^ ^12^ ^]^ Copyright 2016, American Chemical Society. e) Comparison of 23 of 466 calculated colors correlated with experimental data. Metallic (semiconducting) (*n*,*m*) SWCNTs are indicated by the white (black) text in the color plate. f) Distribution of experimentally correlated colors arranged by diameter and chiral angle; most of them are semiconducting CNTs with diameters below 1 nm.

In the aforementioned discussion, we explained the coloration mechanism of SWCNT suspensions and films of both single chirality and of mixed (*n*,*m*). One can modulate the hue of colors by mixing SWCNT samples, though the new color will be less vivid (have lower chroma) than at least one of the parent colors. Because the color range of SWCNT pigments is determined by their most colorful species, and the colorful species (ones with a chroma greater than 50) have small diameters (<2.3 nm) (Figure S5, Supporting Information), we believe that the color map, which covers all (*n*,*m*) below diameters of 2.3 nm, shows the achievable color range for all SWCNT‐based films. Other factors affect the color appearance of SWCNT‐based samples such as the peak width (affected by bundling^[^ ^25^, ^42^ ^]^), relative height of the peaks (affected by dielectric environment and doping), or the intensity of the π‐plasmon peak in the OAS. We thus carried out a sensitivity analysis to determine the influence of each of these factors. Notably, the π‐plasmon peak, located mainly in the ultraviolet region, has more influence (Figures S8 and S9, Supporting Information) on color than the other two minor factors. However, the π‐plasmon peak^[^ ^11^, ^35^, ^43^, ^44^ ^]^ has been poorly studied, and its relationship with SWCNT species has not been reported. It is expected that the structural defects on SWCNTs would also affect the color, but our model in the current shape, unfortunately, has to leave it out for the time being. In this work, the SWCNTs produced from the floating catalyst chemical vapor deposition (FC‐CVD) process contain little defects which have been demonstrated by both high‐resolution TEM observations and Raman measurements.^[^ ^15^, ^25^ ^]^ We believe that further investigating these factors would increase the accuracy of the SWCNT color model.

In conclusion, we have established a systematic quantitative coloration model for understanding the colors of SWCNTs. The new model incorporates theoretical calculations of optical transition energies, a semi‐empirical model for optical absorption, established standards in color science, and our own work on the relationship between OAS and visual color. The resulting coloration model explains the mechanism underlying the coloration of SWCNT films. With the help of the coloration model, we obtained a color map of 466 different species of SWCNTs that discloses the color range of SWCNTs. This study shows that pure carbon atoms arranged in a hexagonal, honeycomb‐like lattice, when shaped into a tubular structure, can produce a full spectrum of strongly colored pigments.

## Experimental Section

### Synthesis of FC‐CVD SWCNTs

SWCNTs were synthesized in an FC‐CVD system based on the thermal decomposition of ferrocene by flowing CO (50 sccm chemical carbon monoxide, 99 vol%, AGA) through a cartridge storing ferrocene. The ferrocene‐containing gas was then injected through a water‐cooled injector probe, which maintained a constant temperature (24 °C). Pure CO was also introduced from the main inlet (250 ccm) and from the bypass inlet (100 ccm) to avoid turbulence and to ensure laminar flow. The ferrocene vapor immediately decomposed into iron vapor after exiting the injector probe and entering the tube furnace (Entech, Sweden), followed by nucleation to iron nanoparticles in the quartz tube at a maximum temperature (850 °C). The iron nanoparticles catalyzed the growth of SWCNTs inside the reactor, and CO_2_ was used to tune the growth of SWCNTs.^[^ ^27^ ^]^ In this work, an additional CO_2_ flow was used (1.0 ccm, controlled by mass flow rate, Aalborg System, USA), corresponding to a volumetric fraction of 0.25 vol%. Finally, the SWCNT network was collected downstream from the reactor for various times using a membrane filter with the help of a vacuum. The resulting SWCNT films were directly press‐transferred onto a quartz slide without any purification for the absorption measurements, as described in further detail in the previous report.^[^ ^15^ ^]^

### Preparation of Solution‐Separated (6,5) SWCNT Films

The aqueous two‐phase method^[^ ^35^ ^]^ was used to enrich (6,5) SWCNTs, and a controlled vacuum filtration method^[^ ^36^ ^]^ was used to fabricate films enriched with (6,5) SWCNTs. To increase the thickness of the film, the films were transferred via the wet‐transfer technique.^[^ ^37^ ^]^ The purity was determined to be 93.2 ± 0.4% by an optical procedure.^[^ ^38^ ^]^

### Measuring Absorbance

The absorption spectra of SWCNT films were measured by a UV–visible–near‐infrared spectrometer (Agilent Cary 5000). In detail, the SWCNT film was transferred from the filter to a quartz substrate. Another empty quartz substrate was used as a reference to avoid the influence of substrate contribution. The wavelengths ranging from 200 to 2600 nm were used. The beam was tuned to a suitable size to fit the sample size.

## Conflict of Interest

The authors declare no conflict of interest.

## Author Contributions

N.W.: Conceptualization, formal analysis, methodology, investigation, visualization, software, data curation, resources, validation, project administration, writing—original draft, writing—review and editing; Y.T.: Methodology, data curation, resources, visualization, writing—review and editing; Y.L.: Resources, investigation, formal analysis, validation, writing—review and editing; N.K.: resources, investigation, formal analysis, writing—review and editing; W.G.: Resources, investigation, writing—review and editing; A.L.: Visualization, funding acquisition writing—review and editing; Q.Z.: Resources, investigation, writing—review and editing; A.H.: Resources, investigation, writing—review and editing; E.‐X.D.: Resources, investigation, writing—review and editing; F.Y.: Methodology, software, formal analysis, writing—review and editing; J.H.: Conceptualization, methodology, formal analysis, investigation, visualization, validation, writing—review and editing; K.L.: Methodology, conceptualization, software, formal analysis, writing—review and editing; J.K.: Methodology, conceptualization, resources, investigation, writing—original draft, writing—review and editing; H.J.: Conceptualization, resources, investigation, visualization, writing—original draft, writing—review and editing; E.I.K.: Funding acquisition, supervision, conceptualization, project administration, writing—review and editing.

## Supporting information

Supporting Information
