# Supplementary material for: Colors of Single‐Wall Carbon Nanotubes
Source: Adv Mater. 2020 Dec 14;33(8):2006395. doi: 10.1002/adma.202006395 (PMC11469110; doi:10.1002/adma.202006395)
Supplement: Supplementary file 1 — Supporting Information [file ADMA-33-2006395-s001.pdf]

© 2020 Wiley-VCH GmbH

ADVANCED MATERIALS

Supporting Information

for *Adv. Mater.*, DOI: 10.1002/adma.202006395

Colors of Single-Wall Carbon Nanotubes

*Nan Wei, Ying Tian, Yongping Liao, Natsumi Komatsu, Weilu Gao, Alina Lyuleeva-Husemann, Qiang Zhang, Aqeel Hussain, Er-Xiong Ding, Fengrui Yao, Janne Halme, Kaihui Liu, Junichiro Kono, Hua Jiang, and Esko I. Kauppinen**

((Supporting Information can be included here using this template))

© 2020 Wiley-VCH GmbH

Supporting Information

Colors of single-wall carbon nanotubes

Nan Wei, Ying Tian, Yongping Liao, Natsumi Komatsu, Weilu Gao, Alina Lyuleeva, Qiang Zhang, Aqeel Hussain, Er-Xiong Ding, Fengrui Yao, Janne Halme, Kaihui Liu, Junichiro Kono, Hua Jiang, and Esko I. Kauppinen*

Methods

Simulating SWCNT film absorbance from (n,m) (The Absorbance sub-model)

A semi-empirical model from previous studies[35,43] was employed to produce the absorption spectra from a specified (*n*,*m*). This model is based on the absorption measurements of suspended individual SWCNTs and can reconstruct the SWCNT absorbance with an accuracy of 20%. This model simulates exciton peaks as well as the asymmetric exciton–phonon sideband (EPS) peaks. For each SWCNT transition (*n*, *m*, *p*), the transition energy is adopted from previous theoretical calculations[27,34] instead of experimental data. The line shape is

simulated according to supplementary equation S6 and supplementary Table S1 from reference[35]

$$\underline{\text{Eq. 1}}\ \frac{\Sigma_p}{\pi}\frac{w_p}{(E-E_p)^2+w_p^2}+\frac{\Sigma_p}{2ab\pi w_p}conv\left(\frac{b\,w_p}{E^2+(b\,w_p)^2},\frac{\mathrm{H}\left(\mathrm{E}-(\mathrm{E_p}+\Delta)\right)}{\sqrt{\mathrm{E}-(\mathrm{E_p}+\Delta)}}\right)$$

where conv(_,_) is the convolution operator $conv(f,g)(E)=\int f(E)g(t-E)dt,$ and the convoluted line shape must be normalized to a maximum of 1; d is the SWCNT diameter; p is an integer indexing optical transitions of both semiconducting and metallic nanotubes starting from 1 in the order of S11, S22, M11, S33, S44, M22, S55, S66, M33, S77, S88, M44, S99, S1010, and M55; Σ_p is $45.9/[(p+7.5)d]$; w_p is $0.0194\,E_p\,f_{pb}$ and $0.0214\,E_p\,f_{pb}$ for semiconducting and metallic SWCNTs, respectively; f_{pb} is the peak broadening factor, which is determined to be 2 for film samples; a is $4.673-0.747\,d$ and $0.976+0.186\,d$ for semiconducting and metallic SWCNTs, respectively; b is $0.97+0.256\,d$ and $3.065-0.257\,d$ for semiconducting and metallic SWCNTs, respectively; and Δ is $0.273-0.041\,d$ and $0.175-0.0147\,d$ for semiconducting and metallic SWCNTs, respectively. Three modifications are applied to the original model. First, a peak broadening factor f_{pb} is added to the original peak width w_p; this factor is determined to be 2 by fitting the presented film absorption data. Second, the first nine transitions of the SWCNT are employed instead of measured transitions between 1.45 to 2.55 eV. The intensity of the EPS sideband is halved to adjust the background in the visible region to the previous level. Third, the wavelength region is extended beyond the original 1.45 to 2.55 eV. To determine the color, the energy range of 1.55–4.13 eV (300–800 nm) is used to convert the absorption cross-section in the original model to the practical film absorbance. A multiplicative factor, $f_a=3.6\times10^{23}\pi d$ cm^{-2} m^{-1}, is applied, assuming the line density of SWCNTs of the same film thickness are always the

same. The whole simulated OAS for Figure 2**a** is routinely red-shifted by 28 meV to account for the dielectric difference between the solution-processed film and the directly synthesized dry film.

Calculating the radiance factor from the absorbance spectra (the Radiance sub-model)

The radiance factor (spectra) in this model, with values between 0 to 1, represents how the SWCNT sample changes the light that illuminates it. It has the same meaning as spectra variation in some other studies.[18] This factor corresponds to the input of the Coordinate sub-model; herein, the same terminology and meaning are adopted as those in the standard for color measurement.[19] The radiance factor is calculated by referring to the definition of absorbance and the experimental results in Figure 2.

<u>Eq. 2</u> $R(\lambda) = 10^{-2A(\lambda)}$

where $A(\lambda)$ is the input absorbance spectra measured from the spectrophotometer or calculated from the Absorbance sub-model.

From radiance factor to color coordinates (the Coordinate sub-model)

The radiance factor of the SWCNT sample is converted to color coordinates in various standard color spaces according to the standard.[19]

In the standard procedure, the radiance factor is first converted to XYZ color coordinates using equations 3 to 5.

<u>Eq. 3</u> $X = k \int R(\lambda)S(\lambda)\, \bar{x}(\lambda)\, d\lambda$

<u>Eq. 4</u> $Y = k \int R(\lambda)S(\lambda)\, \bar{y}(\lambda)\, d\lambda$

<u>Eq. 5</u> $Z = k \int R(\lambda)S(\lambda)\, \bar{z}(\lambda)\, d\lambda$

where $R(\lambda)$ is the input radiance factor, and $S(\lambda)$ is the standard illuminant. In this report, we chose the standard illuminant D65, which corresponds to noon daylight and is the standard light source in many RGB standards.[17] The normalization factor, *k*, is given by:

Eq. 6 $k = 100/\int S(\lambda)\,\bar{y}(\lambda)\,d\lambda$

This factor normalizes the XYZ values so that a completely white object, which has all one radiance factor under one illuminant, will have the XYZ coordinates of (100,100,100). $\bar{x}(\lambda)$, $\bar{y}(\lambda)$, and $\bar{z}(\lambda)$ are the color matching functions of one of the CIE standard observers. In this study, the CIE 1931 2° standard observer was adopted, which corresponds to the average human light vision. In this way, the result of this report can be compared with established standards in the reference.[16,17] In programming implementation, the Python library color-science[20] is employed to convert the radiance factor to XYZ color coordinates as well as to convert between different color coordinates (mainly between XYZ, La*b*, L*C*h, and sRGB).

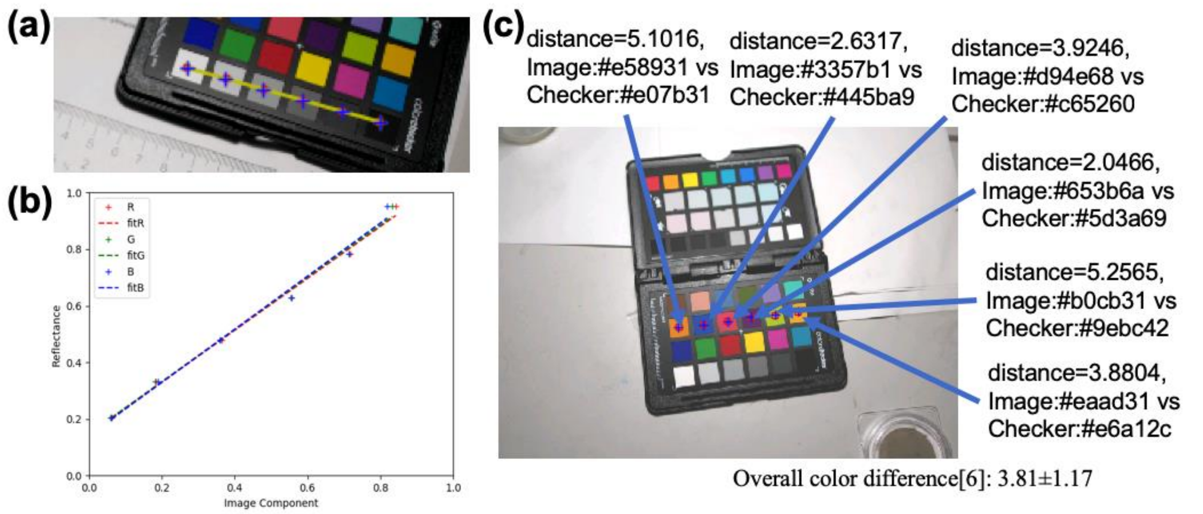

Figure S1. Calibration of photographic images

To calibrate a photographic image, and to obtain more accurate colors than the ones gained with the human eye, a single light source with a high color rendering index ($R_A > 95$) is used, and the uniformity of the lighting is ensured. **a**, A photograph taken with a standard color palette (herein, the X-rite ColorChecker Passport is used). A moderate exposure level was adopted that did not overexpose the white patch or underexpose the black patch. The photograph should be taken using the camera RAW format. The standard calibration process, as indicated in the color standard, was performed to adjust the exposure and white balance. **b**, In order to recover the absolute color coordinates, an additional fitting to the RGB color coordinates[18] was applied to the sampled color values of the gray patches of the color standard. Then, the fitting parameters were applied to the whole image to obtain the calibrated photograph. **c**, The result was verified against nominal values from the standard color palette.[17] A complete verification against all 24 color patch values revealed a color difference, ΔE, of 3.0 ± 1.5. This difference was below the threshold of 5, common for a general human eye to recognize color difference.[16]

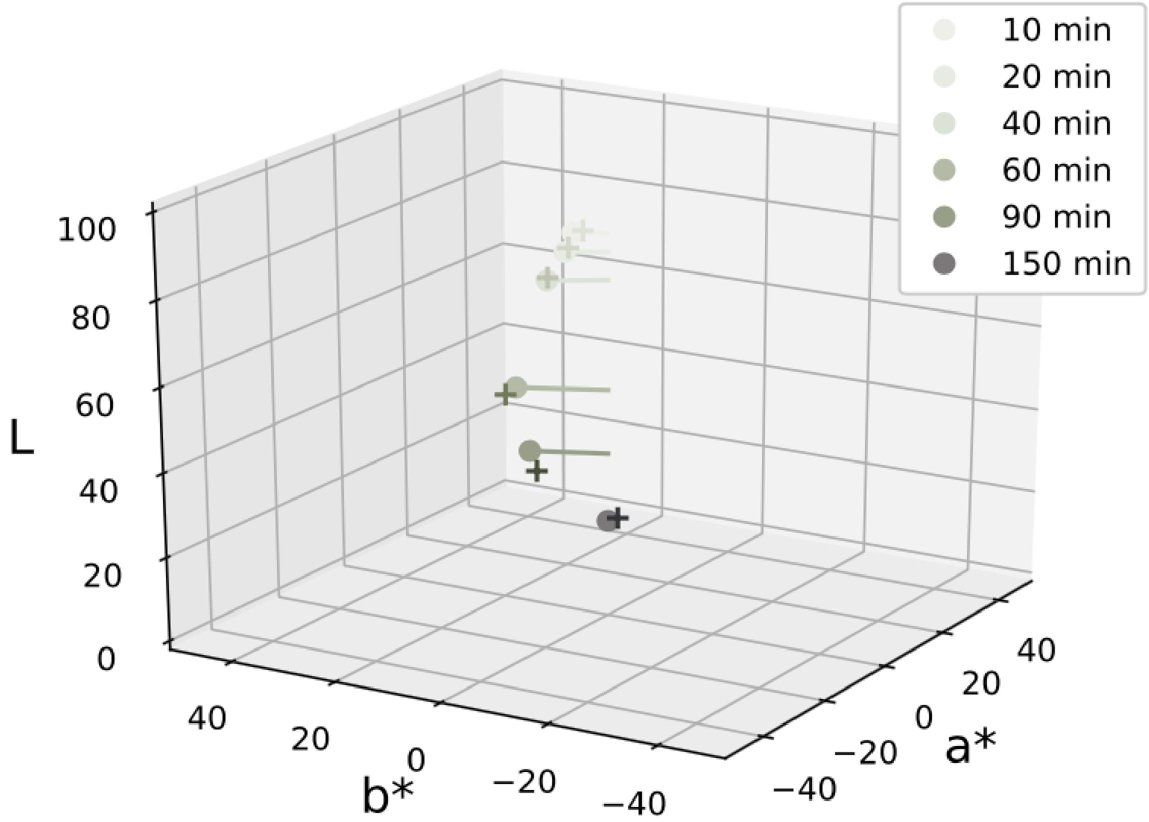

Figure S2. Perspective 3D plot of calculated and measured color coordinates.

The L*a*b* color space is a standard for representing color difference,[16,17] and it is closely related to the definition of hue and chroma. The Euclidean distance and angle in the a*b* plane define the chroma and hue, respectively.[16] For clarity, the main text shows only two-dimensional profiles of the six datasets. This figure shows a perspective view of the three-dimensional plot.

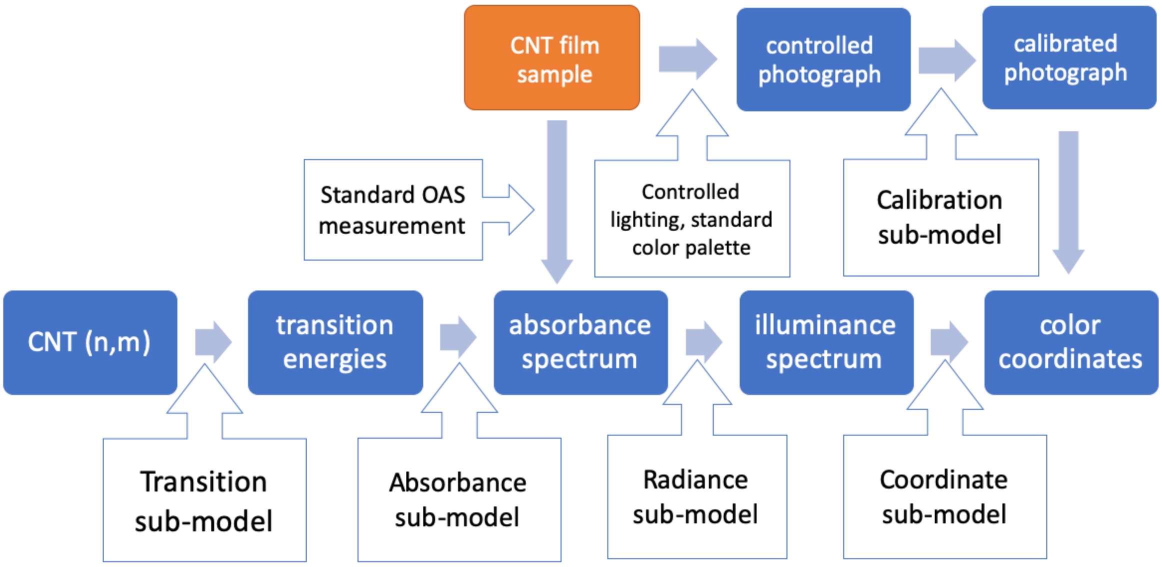

Figure S3. Sections and process flow of the coloration model.
The overall model has four internal sections that process five types of data. The connection between these sections is shown. In the practical application of this overall model, the extended tight binding calculation with many-body corrections is employed as a database (the Transition sub-model), whereas the calculations for other steps are performed sequentially. The algorithms of these sections can be independently substituted (modularized) to increase the accuracy of the overall model. The spectral integration process in Section 4 (the Coordinate sub-model) is an established standard process.[19] Section 3 (Radiance sub-model) follows a simple ink model that is not optimized further. However, Section 2 (Absorbance sub-model) offers some room for improvement; more experimental data can be obtained.

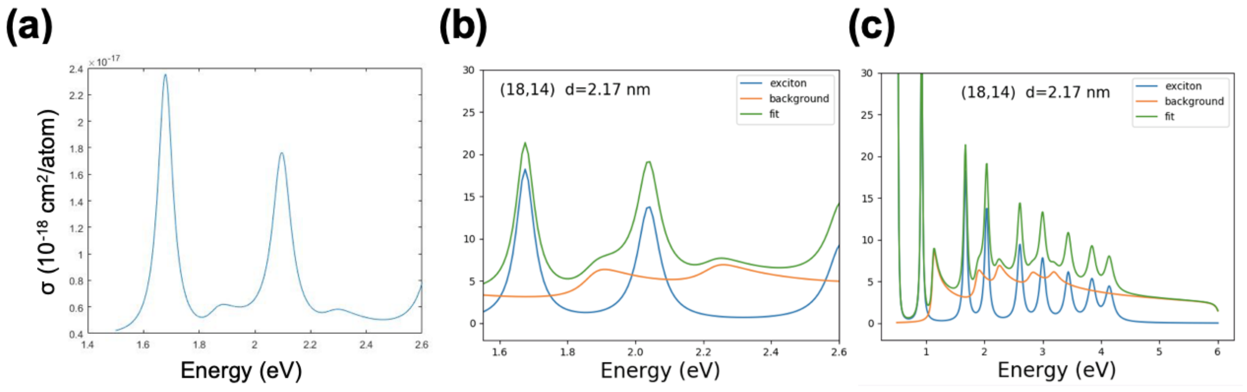

Figure S4. Simulated absorbance spectra compared with Liu's original model.

In order to calculate colors, Liu's original model for the absorption cross-section of SWCNTs is employed with minor modification. **a,** is extended to fit into the overall model. The resulting Absorbance sub-model, **b**, accepts transition energies of SWCNTs up to the ninth transition and doubles the width of all excitonic peaks to better fit the film data. Additionally, it is extended to a wider spectral range, **c**, than the original 500–800 nm. The unit of the output spectra is converted from the original absorption cross-section (cm^2 per atom) to the absorbance of the film (cm^{-1}). The assumption of this conversion is the line densities of SWCNT films of the same thickness are the same among different (*n*,*m*). The background includes all exciton–phonon sidebands of all the transitions. Thus, the extended version remains true to the original model in the former spectral range of 500–800 nm. Additionally, it has extended variability by accepting more transitions and has a broader spectral range,

leading to a better fit for calculating the colors.

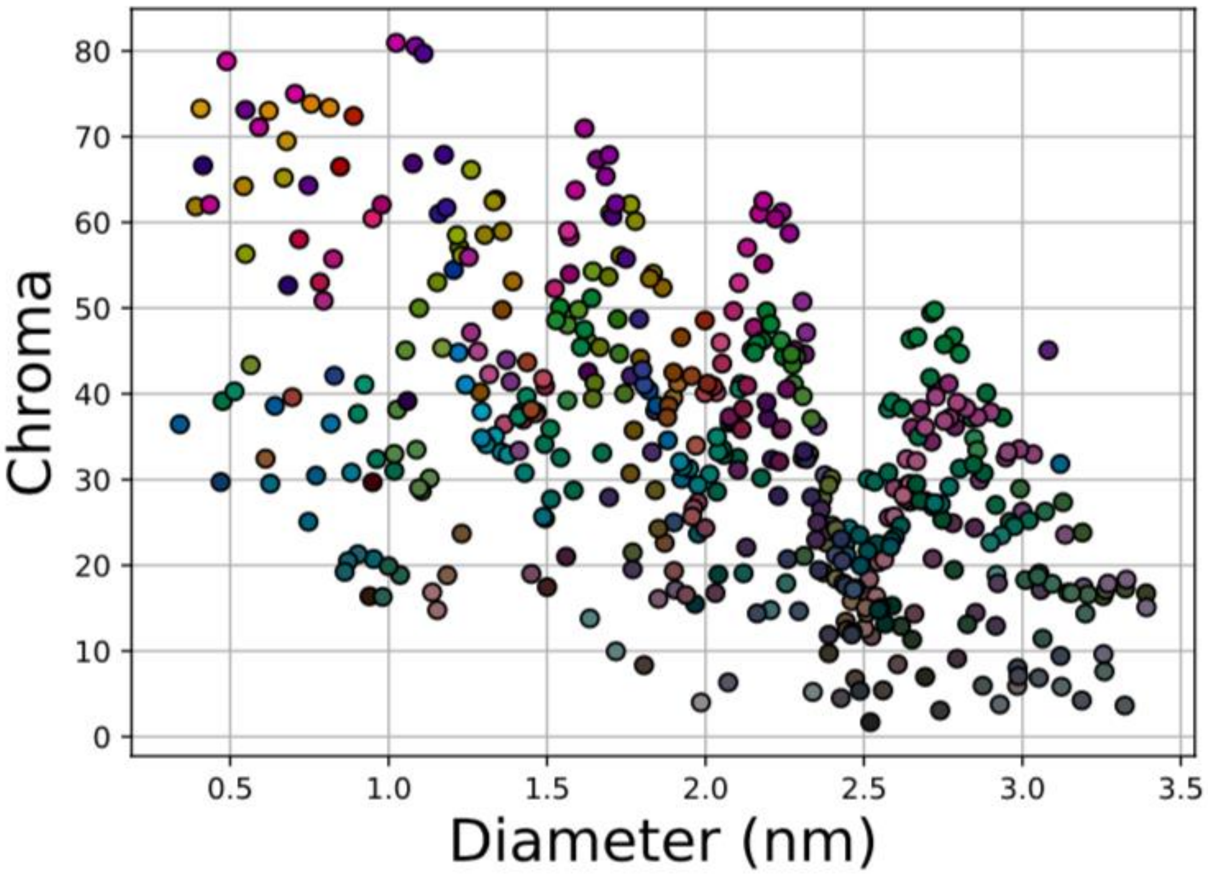

Figure S5. Relationship between diameter and chroma of SWCNTs.
The most vivid colors of various (*n*,*m*) SWCNTs were calculated by the coloration model. The chroma components of the colors were extracted and plotted against the diameter of the corresponding SWCNTs. The plot shows that as the diameter increases, SWCNTs tend to have lower chroma, i.e., the color is closer to gray. This trend is in concert with the fact that the properties of large-diameter SWCNTs would approach those of graphene.

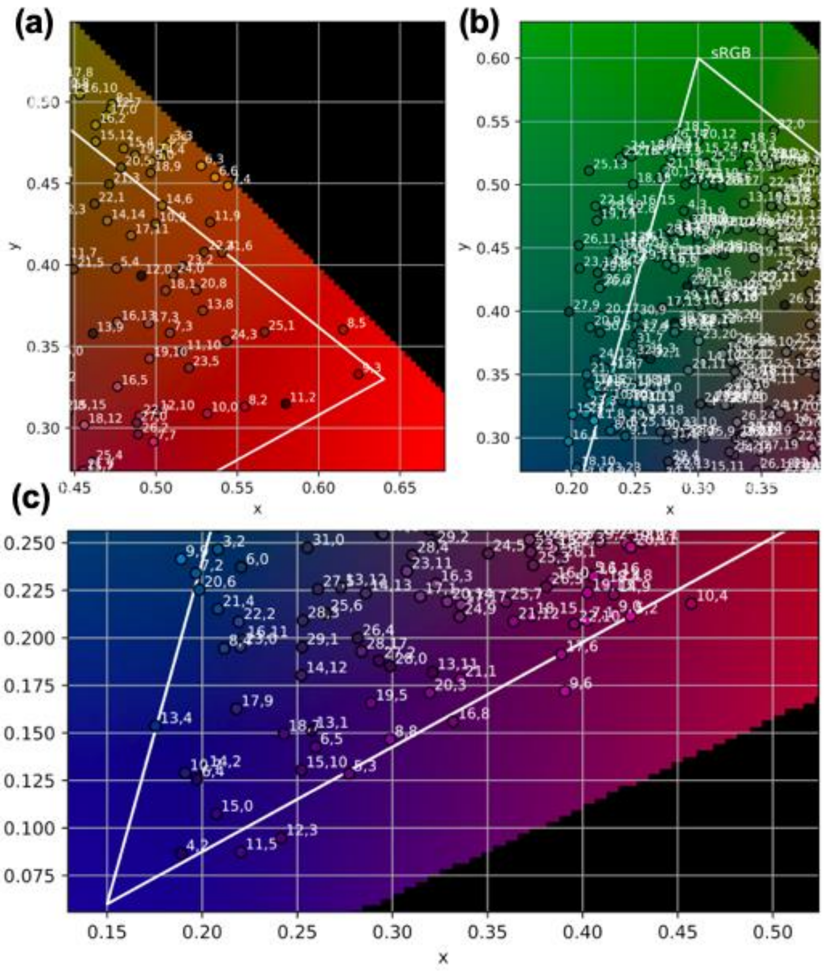

Figure S6. Extreme colors among SWCNTs.
Enlarged parts of Figure 1B are shown with indicated (*n*,*m*). The most colorful species of SWCNTs can be identified. **a,** Red and yellow region. Candidates for most red SWCNTs are (9,3) and (8,5) and for most yellow are (6,3), (6,6), and (7,4). **b,** Green and cyan region. Candidates for the most green SWCNTs are (18,5) and (22,0) and for most cyan are (15,3), (24,1), and (11,8). **c,** Blue and magenta region. Candidates for the most blue SWCNTs are (4,2) and (15,0) and for most magenta are (9,6), (17,6), and (22,10).

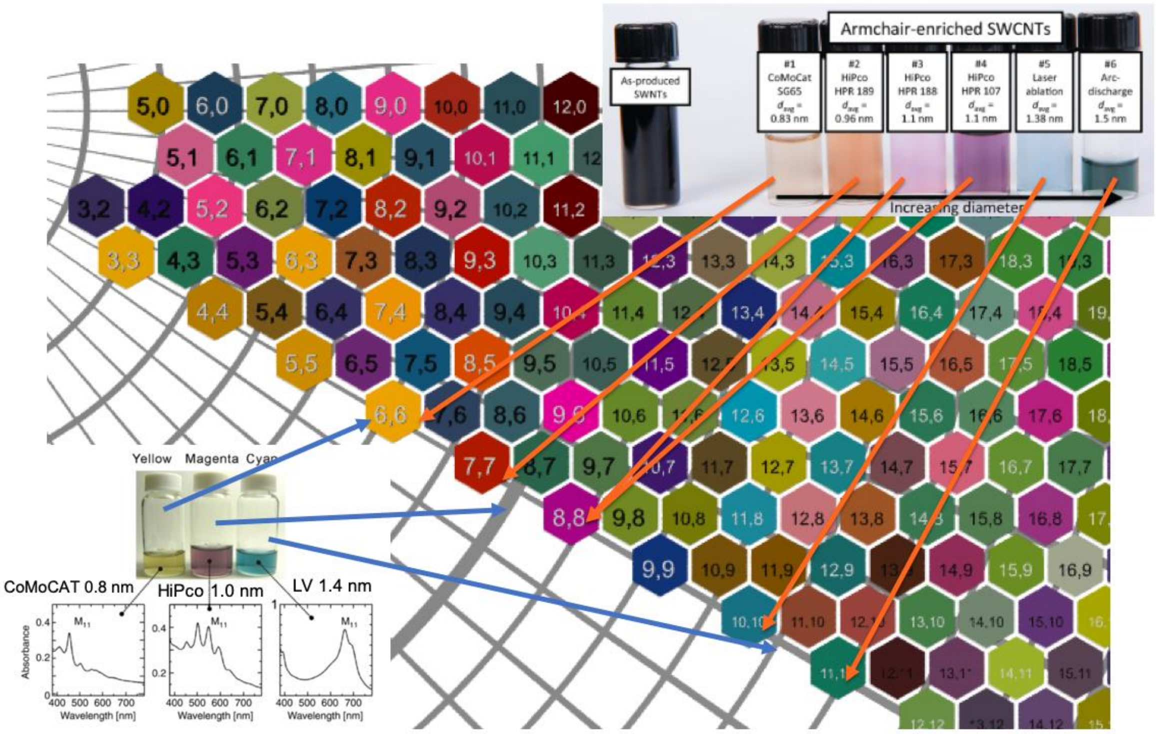

Figure S7. Explaining multiple-(*n*,*m*) results using the color map.

The color map generated by the coloration model can also explain the color of multiple-(n,m) SWCNT-based samples. For example, though the results from ref. [10] (upper right) contains multiple armchair (*n*,*m*) species, the colors can be explained by mixing the nearby colors of the indicated diameter. For example, the color of the suspension of 0.96-nm-diameter SWCNTs can be achieved by mixing the colors given by (6,6), (7,7), and (8,8) SWCNTs. The same explanation can be applied to another enriched metallic SWCNT-based sample[7] (lower left).

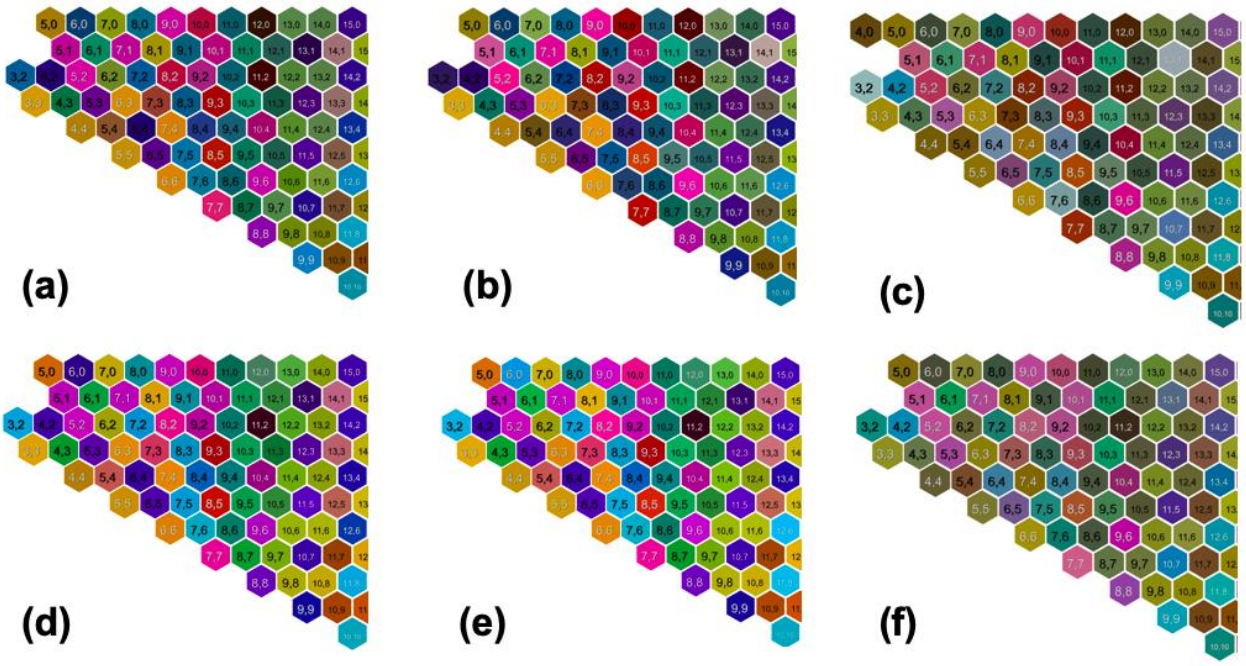

Figure S8. Results of the sensitivity analysis of adopting different Absorbance sub-models.

(a-c) Liu's model[35] with **a)** 2× broader peaks for film samples, which is adopted in the present coloration model, **b)** the original peak width, which is closer to suspended SWCNTs, and **c)** with the original peak and a plasmon peak with the intensity of the green film sample.[15] **(d-f)** Uniform Lorentzian peaks with **d)** a full width at half maximum (FWHM) of 200 meV, **e)** a FWHM of 100 meV, and **f)** a FWHM of 100 meV with the same plasmon peak as the green film. Increasing peak widths from suspended SWCNTs to SWCNT films generally lead to more saturated color, while plasmon peaks lead to more green-yellowish colors. However, both variations have limited influence on the resulting colors. For example, blue, green, and red species do not change to other colors. Thus, in this case, minor changes in the Absorbance sub-model do not change the main result. (**a)** serves as the main model in the present work, because the height and width of its absorbance peaks best match the experimental data of the SWCNT films.

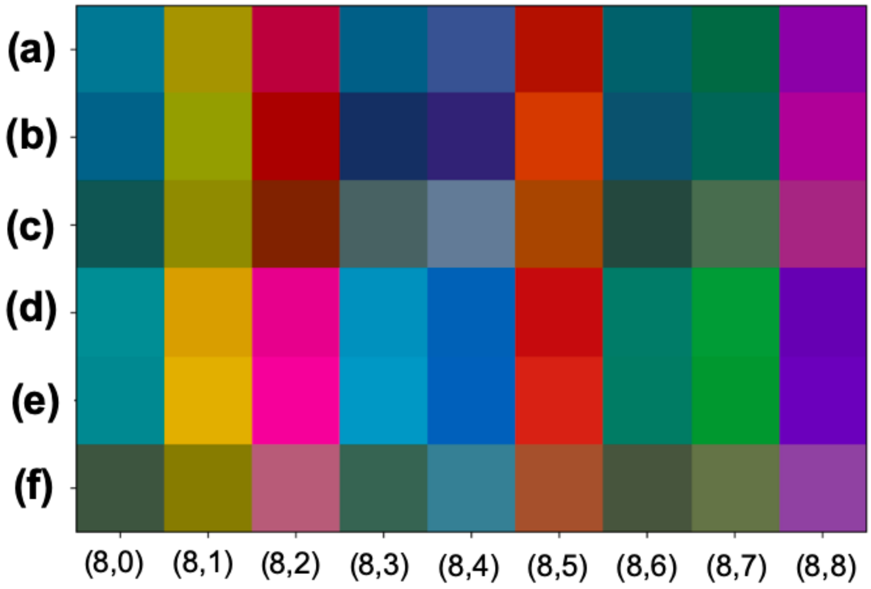

Figure S9 | Color matrix of the sensitivity analysis of adopting different Absorbance sub-models.
The color matrix shows a specific set of colors (most vivid colors of SWCNTs with n = 8) from Figure S8 for a closer comparison. (**a–c)** Liu's model with **a)** 2× broader peaks for film samples, which is adopted in the present coloration model, **b)** the original peak width, which is closer to suspended SWCNTs, and **c)** with the original peak and a plasmon peak with the intensity of the green film sample. (**d–f)** Uniform Lorentzian peaks with **d** a FWHM of 200 meV, **e** a FWHM of 100 meV, and **f)** a FWHM of 100 meV with the same plasmon peak as the green film. Evidently, the lightness and chroma of the colors are more sensitive to the assumptions, while the hues are less sensitive. Thus, the predicted hues are stable, despite variations in the assumptions of the peak width and existence of a plasmon peak.

Table S1. Measured and calculated color coordinates of the films with various thicknesses

Sample collection duration (min)	Calculated coordinates from absorbance	From absorbance with specular correction	Measured coordinates from photo
10	87, −2.2, 5.3	88, −2.2, 5.3	89, −1.6, 3.7
20	83, −3.7, 6.5	84, −3.5, 6.0	85, −2.7, 5.8
40	76, −5.4, 8.3	78, −5.3, 8.3	78, −4.2, 8.8
60	48, −7.2, 15.3	52, −6.4, 13.6	50, −6.4, 15.6
90	30, −6.7, 16.9	37, −5.2, 11.8	32, −4.3, 11.0
150	0, 0, 0	21, 0, 0	22, −0.2, -2.0

The SWCNT films are photographed directly after being collected on the filter membrane. Subsequently, the films are transferred to quartz glass slides for the absorbance measurement. Colors can be calculated from the measured absorbance spectra using the model proposed herein (the latter two steps of the coloration model). In practice, the color of the samples has a specular (mirror reflection) term because the films are optically flat (i.e., the film surface roughness is much smaller than optical wavelengths). Even though the specular image of the light source is excluded from the camera's detection cone, a specular correction is still necessary because the environment is not totally black, and the non-linear response of human eye is sensitive to changes in dark tones.[21] This single value term is obtained by fitting a common white light component to the calculated color of six samples. After this specular

correction, all of the color coordinates match within an error range ΔE < 6. This agrees with the measured error range for the presented photographic color calibration method.
